# Biological properties of novel ruthenium- and osmium-nitrosyl complexes with azole heterocycles

**DOI:** 10.1007/s00775-016-1345-z

**Published:** 2016-03-09

**Authors:** Maria S. Novak, Gabriel E. Büchel, Bernhard K. Keppler, Michael A. Jakupec

**Affiliations:** Institute of Inorganic Chemistry, University of Vienna, 1090 Vienna, Austria; Division of Physical Sciences and Engineering, KAUST Catalysis Center, King Abdullah University of Science and Technology, Thuwal, 23955-6900 Saudi Arabia

**Keywords:** Ruthenium, Nitrosyl complexes, Cancer, Apoptosis, cGMP level

## Abstract

Since the discovery that nitric oxide (NO) is a physiologically relevant molecule, there has been great interest in the use of metal nitrosyl compounds as antitumor pharmaceuticals. Particularly interesting are those complexes which can deliver NO to biological targets. Ruthenium- and osmium-based compounds offer lower toxicity compared to other metals and show different mechanisms of action as well as different spectra of activity compared to platinum-based drugs. Novel ruthenium- and osmium-nitrosyl complexes with azole heterocycles were studied to elucidate their cytotoxicity and possible interactions with DNA. Apoptosis induction, changes of mitochondrial transmembrane potential and possible formation of reactive oxygen species were investigated as indicators of NO-mediated damage by flow cytometry. Results suggest that ruthenium- and osmium-nitrosyl complexes with the general formula (indazolium)[*cis*/*trans*-MCl_4_(NO)(1*H*-indazole)] have pronounced cytotoxic potency in cancer cell lines. Especially the more potent ruthenium complexes strongly induce apoptosis associated with depolarization of mitochondrial membranes, and elevated reactive oxygen species levels. Furthermore, a slight yet not unequivocal trend to accumulation of intracellular cyclic guanosine monophosphate attributable to NO-mediated effects was observed.

**Electronic supplementary material:**

The online version of this article (doi:10.1007/s00775-016-1345-z) contains supplementary material, which is available to authorized users.

## Introduction

**Fig. 1:**
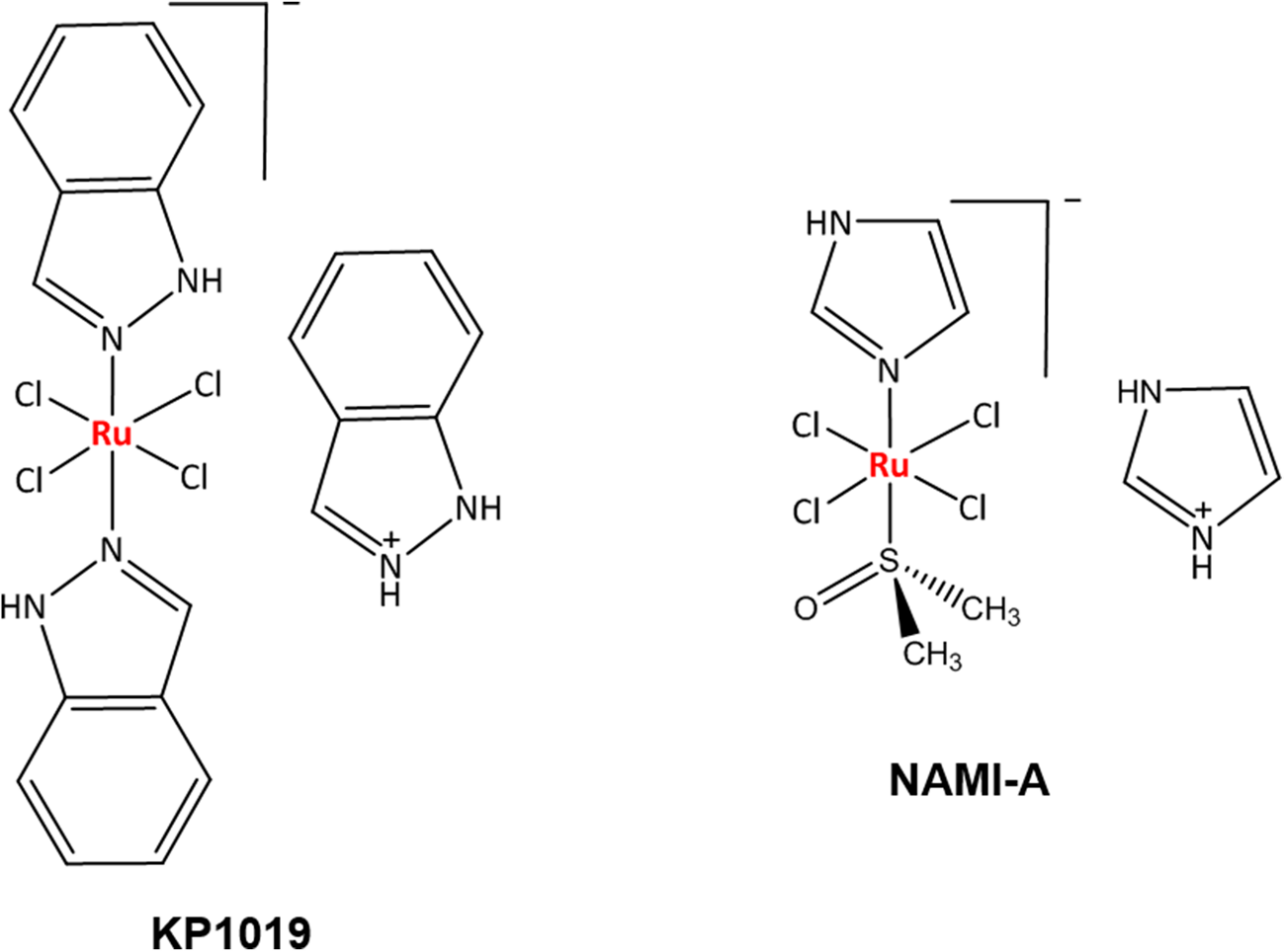
Structures of indazolium *trans*-tetrachloridobis(1*H*-indazole)ruthenate(III) (KP1019) and imidazolium *trans*-tetrachlorido(dimethylsulfoxide)imidazoleruthenate(III) (NAMI-A)

Efforts of many scientists around the world have been focused on the design of metal-based agents that can be successfully used in cancer therapy with a proper balance between activity and toxicity profiles, being active against cancer cells, but not too harmful to normal cells. Ruthenium-based complexes show great promise, not only because of their lower toxicity compared to some other metals, but also because of different mechanisms of action and a different preference for protein rather than DNA binding in comparison to platinum drugs and a different spectrum of activity without pronounced cross-resistance [1, 2]. Over time, several ruthenium complexes were developed and studied for their antiproliferative activities against various cancer models, with the clinical development of NAMI-A, KP1019 (Fig. 1) and NKP-1339 being the major milestones in this field of research. NAMI-A shows little activity against primary tumors, but was found to be highly active against secondary tumors, particularly lung metastases, while the activity of KP1019 affects primary tumors as well, such as colorectal cancers which are resistant to cisplatin therapy [3–8]. Recently, NKP-1339 has been studied against solid tumors and showed promising results in a phase I clinical trial, most remarkably in patients with gastrointestinal neuroendocrine tumors [9]. It was suggested that the tumor targeting properties of KP1019 and NKP-1339 are based on their reversible binding to serum proteins [10–12].

Nitric oxide releasing (pro)drugs of an organic (nitroglycerin) and inorganic (sodium nitroprusside) kind were already in clinical use for decades before the discovery that such a small molecule could act as a signaling molecule in biological systems [13–15]. In recent years, several classes of exogenous NO donors have been synthesized with the aim to investigate physiological processes controlled by different concentrations of nitric oxide in the cell. Some research groups studied the photochemistry of such compounds, and various strategies were developed to activate these compounds to release NO under the influence of visible or UV/IR light [16]. Generally, ruthenium nitrosyl complexes are attractive because of the thermal stability of the Ru–NO bond, which upon photochemical or electrochemical stimulation may release NO [17–21]. Besides this, it was proposed that the high affinity of NO to ruthenium might play a role in the mechanism of action of KP1019, which may act as an NO scavenger.

**Fig. 2:**
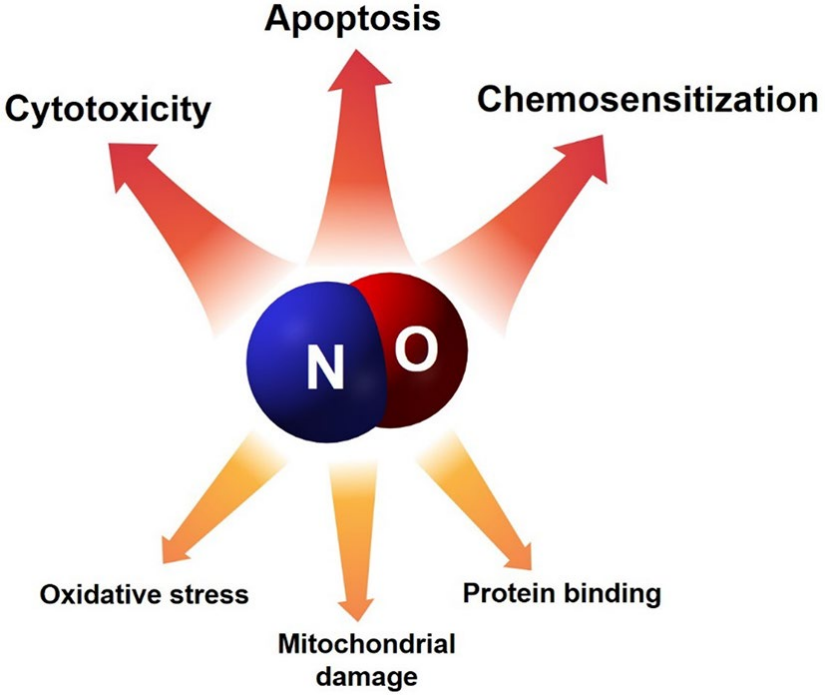
Nitric oxide is highly reactive molecule that is able to regulate a wide range of cell physiological processes

Nitric oxide is a highly reactive free radical containing an unpaired electron in its outermost orbital, allowing it to take part in many different reactions as an electron donor or acceptor and is capable of enhancing various reactions and processes (Fig. 2). Thereby, NO is able to react with other inorganic molecules, DNA, prosthetic groups or with proteins [22]. As a ligand in inorganic chemistry, NO is also known as a “non-innocent ligand”, implying that NO can adopt several oxidation states rendering the assignment of oxidation states for the ligand and the metal complicated. Furthermore, the geometry of NO bound to a metal can vary largely from being bound linearly to strongly bent, and being coordinated via N, O or both atoms at the same time. In biological systems, nitric oxide is an endogenous molecule that is produced by nitric oxide synthase (NOS) through the conversion of l-arginine to l-citrulline and NO [23]. Additionally, NOS-independent NO generation pathways starting from nitrate (NO_3_^−^) and nitrite (NO_2_^−^), previously thought to be inert, were discovered recently [24]. Activation of soluble guanylate cyclase (sGC), formation of cGMP, and concomitant protein phosphorylation is considered the main physiological signaling pathway of NO. This cGMP-dependent pathway activates various downstream targets including protein kinases, phosphodiesterases and ion channels; whereas cGMP-independent pathways can be related to *S*-nitrosylation, which leads to an inhibition of mitochondrial cytochrome oxidase. The physiological functions of NO include blood pressure control, neurotransmission, immunological responses, and antioxidant defense. All these effects are strongly dependent on the local NO bioavailability and concentration—while low concentrations of nitric oxide contribute to host defense, high concentrations may promote apoptosis [1, 20, 25]. The administration of NO donors reduces NF-kB activation and downstream expression of anti-apoptotic gene products, which is relevant for NO-dependent sensitization of chemotherapy-resistant tumor cells [26–28]. Mitochondrial damage during NO-mediated apoptosis could be linked to the decrease of mitochondrial transmembrane potential due to the opening of mitochondrial permeability transition pores followed by massive ROS production [29].

**Fig. 3:**
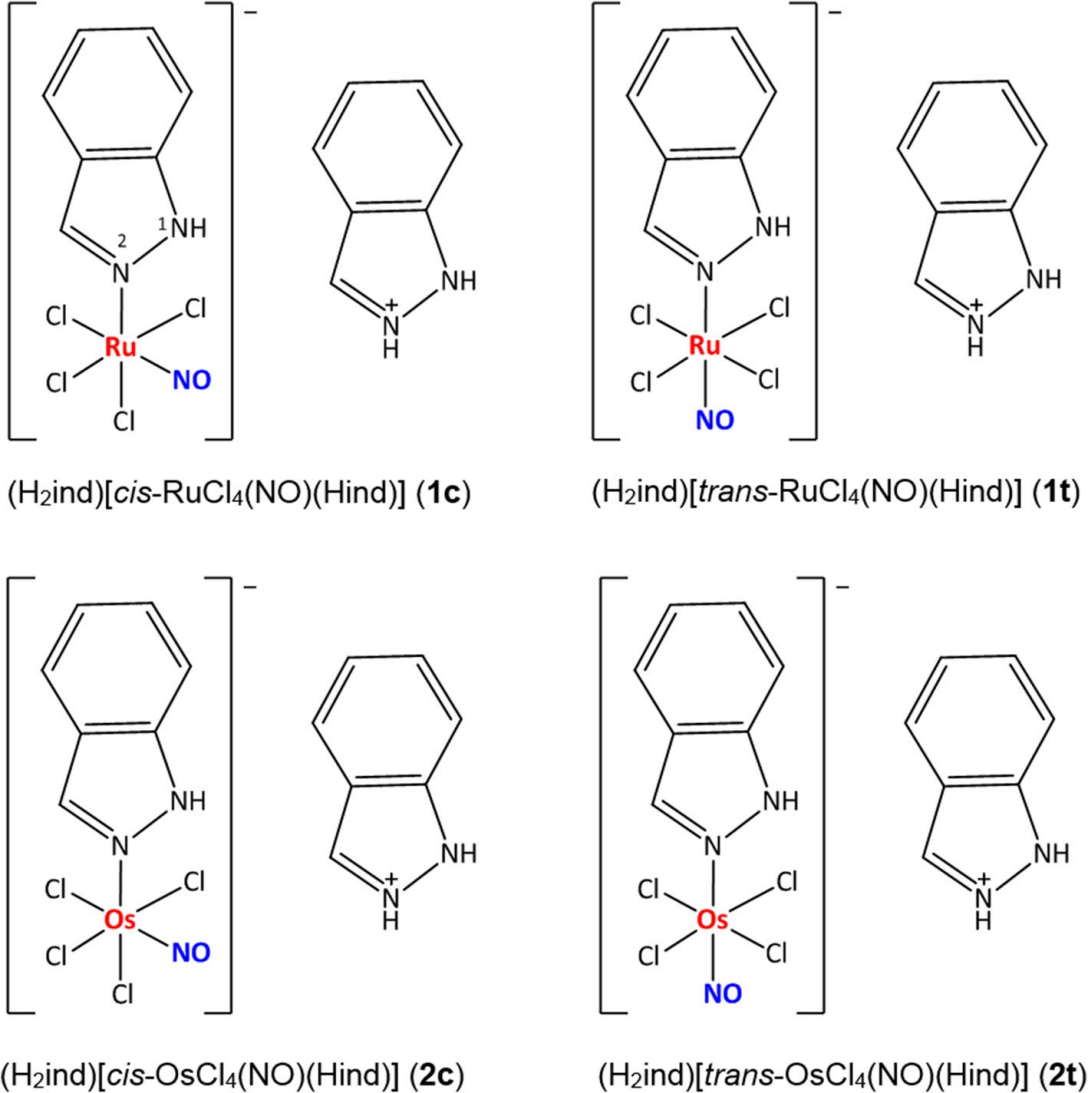
Structures of ruthenium- and osmium-based nitrosyl complexes. Nitrogen atom labeling was introduced in **1c**

To characterize in more detail the biological features of ruthenium- and osmium-nitrosyl complexes with the general formula (indazolium)[*cis/trans*-MCl_4_(NO)(1*H*-indazole)] (Fig. 3), we report here on cytotoxicity tests, flow cytometric detection of mitochondrial membrane depolarization, ROS generation and apoptotic cells, measurement of intracellular cGMP levels as well as plasmid DNA interaction studies. Synthesis, physicochemical measurements and stability in aqueous solutions as well as reactivity toward ascorbic acid, ubiquitin and myoglobin of these complexes have been published previously [30]. It has also be shown by crystallographic structure determination that indazole binds only via N2 to the metal centers and not via N1 as recently found in particular for osmium compounds, an effect also influencing biological properties [31, 32]. X-ray crystallography also reveals that NO is bound linearly via the nitrogen atom, as already indicated by IR spectroscopic data as well as calculations [33]. Potentially biologically relevant *cis*/*trans* isomerization reactions of these complexes were studied, revealing lower activation barriers for ruthenium than osmium compounds [34].

## Materials and methods

### Cell lines and culture conditions

CH1/PA-1 cells (identified via STR profiling as PA-1 ovarian teratocarcinoma cells by Multiplexion, Heidelberg, Germany; compare Ref. [35]) were obtained from Lloyd R. Kelland, CRC Centre for Cancer Therapeutics, Institute of Cancer Research, Sutton, UK. SW480 (human adenocarcinoma of the colon), A549 (human non-small cell lung cancer) and HL-60 (human promyelocytic leukemia) cells were kindly provided by the Institute of Cancer Research, Department of Medicine I, Medical University of Vienna, Austria. All cell culture media and supplements were purchased from Sigma-Aldrich, Austria and plastic ware from Starlab, Germany. Cells were grown in 75 cm^2^ culture flasks in complete medium (i.e., Minimum Essential Medium supplemented with 10 % heat-inactivated fetal bovine serum, 1 mM sodium pyruvate, 4 mM l-glutamine and 1 % non-essential amino acids from 100× ready-to-use stock) as adherent monolayer cultures. Cultures were grown at 37 °C under a humidified atmosphere containing 5 % CO_2_ and 95 % air.

### MTT assay

Antiproliferative activity in vitro was determined by the colorimetric MTT assay (MTT = 3-(4,5-dimethyl-2-thiazolyl)-2,5-diphenyl-2*H*-tetrazolium bromide). For this purpose, cells were harvested from culture flasks by use of trypsin and seeded in complete medium (100 µL/well) into 96-well plates in densities of 3 × 10^3^ (A549), 1 × 10^3^ (CH1/PA-1) and 2 × 10^3^ (SW480) cells per well. Cells were allowed for 24 h to settle and resume proliferation. Test compounds were dissolved in DMSO first, appropriately diluted in complete medium and instantly added to the plates (100 µL/well), where the DMSO content did not exceed 0.5 %. After exposure for 96 h, the medium was removed and replaced with 100 μL/well of a 1:7 MTT/RPMI 1640 solution (MTT solution, 5 mg/mL of MTT reagent in phosphate-buffered saline; RPMI 1640 medium, supplemented with 10 % heat-inactivated fetal bovine serum and 4 mM l-glutamine) and incubated for 4 h at 37 °C. Subsequently, the MTT/RPMI 1640 solution was removed, and the formazan product formed by viable cells was dissolved in DMSO (150 µL/well). Optical densities were measured with a microplate reader (BioTek ELx808) at 550 nm (and a reference wavelength of 690 nm) to yield relative quantities of viable cells as percentages of untreated controls, and 50 % inhibitory concentrations (IC_50_) were calculated by interpolation. Evaluation is based on at least three independent experiments with triplicates for each concentration level.

### Neutral red uptake assay

Additionally, cell viability in vitro was investigated by the neutral red uptake assay (neutral red = 3-amino-7-dimethylamino-2-methylphenazine hydrochloride, Sigma). A549, CH1/PA-1 and SW480 cells were seeded in complete medium (100 µL/well) into 96-well plates in the same densities as for MTT tests and were allowed for 24 h to settle and resume proliferation. Test compounds **1c** and **1t** were dissolved in DMSO first, appropriately diluted in complete medium and instantly added to the plates (100 µL/well), where the DMSO content did not exceed 0.5 %. After expose for 96 h, medium was replaced with 100 µL/well of a neutral red-containing medium. A neutral red stock solution (5 mg/mL) was prepared in phosphate-buffered saline, then diluted 1:100 in RPMI 1640 medium (supplemented with 10 % heat-inactivated fetal bovine serum and 4 mM l-glutamine) and pre-incubated overnight at 37 °C. Plates were incubated with this neutral red solution for 2 h at 37 °C to allow for the uptake into the lysosomes of viable cells. After incubation the medium was removed, cells were washed with phosphate-buffered saline two times (150 µL/well) and fixed with 1 % acetic acid in 70 % ethanol (150 µL/well). After incubation for 10 min at RT, the absorption was measured with a microplate reader (BioTek ELx808) at 550 nm (and a reference wavelength of 690 nm) to yield relative quantities of viable cells as percentages of untreated controls, and 50 % inhibitory concentrations (IC_50_) were calculated by interpolation. Evaluation is based on at least three independent experiments with triplicates for each concentration level.

### Impact on mitochondrial membrane potential (JC-1 assay)

Impairment of mitochondrial transmembrane potential was studied by flow cytometry using the lipophilic cationic dye 5,5′,6,6′-tetrachloro-1,1′,3,3′-tetraethylbenzimidazolyl-carbocyanine iodide (JC-1). For this purpose, SW480 cells were exposed to test compounds in different concentrations for 48 h at 37 °C and then collected in a density of 2 × 10^5^ cells/mL by centrifugation at 500*g* for 5 min. Afterwards, cells were washed with PBS and stained with 2 µg/mL JC-1 mix in complete medium for 15 min in the dark at 37 °C. Then cells were washed and suspended in 500 µL of warm PBS and analyzed with a Guava 8HT EasyCyte flow cytometer (Millipore) using InCyte software. Carbonyl cyanide 3-chlorophenylhydrazone (CCCP) was used as a positive control in a concentration of 0.9 mM.

### Flow cytometric detection of apoptotic cells

Induction of cell death was analyzed by flow cytometry using FITC-conjugated annexin V (BioVision, USA) and propidium iodide (PI, Fluka) double staining. SW480 cells were seeded into 12-well plates in a density of 5 × 10^4^ cells per well in complete medium and allowed to settle for 24 h. The cells were exposed to test compounds in different concentrations for 48 h at 37 °C. The platinum complex KP1988 (synthesized at the Institute of Inorganic Chemistry, University of Vienna) was used as a positive control in a concentration of 200 μM. After incubation, cells were gently trypsinized, washed with PBS, and suspended with FITC-conjugated annexin V (0.25 μg/mL) and PI (1 μg/mL) in binding buffer (10 mM HEPES/NaOH pH 7.4, 140 mM NaCl, 2.5 mM CaCl_2_) at 37 °C for 15 min. Stained cells were analyzed with a Guava 8HT EasyCyte flow cytometer (Millipore) using InCyte software.

### Detection of intracellular reactive oxygen species (ROS)

For the fluorimetric analysis of ROS, non-adherent HL60 cells (promyelocytic leukemia, human) were stained for 30 min at 37 °C under 5 % CO_2_ with 1 μM DCF-DA (2′,7′-dichlorofluorescein diacetate) in Hanks’ Balanced Salt Solution supplemented with 1% heat-inactivated fetal bovine serum. Cells were transferred into 96-well plates in a density of 6 × 10^4^ cells/well and treated with the test substances at different concentrations for 30 min at 37 °C under 5 % CO_2_. A freshly prepared 500 μM H_2_O_2_ solution was used as a positive control and added 10 min before measurement. Cellular ROS levels were measured by flow cytometry on a Guava 8HT EasyCyte flow cytometer (Millipore). The resulting histograms of green fluorescence were quantified by FlowJo software (Tree Star). Results are presented as the ratios of green fluorescence intensities of the drug-treated samples and that of the untreated control.

### Competition enzyme-linked immunoassay (cGMP assay)

The intracellular cGMP levels after treatment with nitrosyl complexes were assessed by using the Cyclic GMP XP™ Assay Kit (Cell Signaling Technology). The teratocarcinoma cell line CH1/PA-1 was grown in 12-well plates under standard conditions and treated with various concentrations of test compounds for 2 h. Then, cells were solubilized in lysis buffer, and intracellular cGMP levels were assessed according to manufacturer’s instructions. The absorbance was measured with a microplate reader (BioTek ELx808) at 450 nm, and the absolute amount of cGMP in samples was calculated by using a standard curve. Evaluation is based on at least three independent experiments with duplicates for each concentration level.

### Plasmid DNA interaction studies

pUC19 DNA (2686 bp) plasmid was purchased from Fermentas Life Sciences. 500 ng of pUC19 plasmid was incubated with 50 μM of the test compounds in 0.1× Tris-EDTA (TE) buffer for different time intervals (5 min up to 6 h) at 37 °C. The electrophoresis was performed in agarose (from Sigma-Aldrich) gel 1 % w/v in 1× Tris-borate-EDTA (TBE) buffer for 90 min at 80 V. Gels were stained with ethidium bromide (EtBr) in 1× TBE (0.75 μg/mL) for 20 min. Images were taken with the multi-imaging detection system Fusion SL (Vilber Lourmat).

## Results and discussion

As reported previously, ruthenium complexes **1c** and **1t** yielded IC_50_ values in the low micromolar range and turned out to be much more cytotoxic than osmium complexes **2c** and **2t** (Table 1). Since the aqueous solubility of these complexes at 294 K is in the very low mM range, compounds had to be dissolved in DMSO, but were diluted to acceptable DMSO contents to enable the application of up to very high submillimolar concentrations in biological test. According to UV-vis spectroscopy (and ESI-MS) studies, complexes remain intact in aqueous solution for at least 24 h (Ru) and 72 h (Os), respectively. ESI-MS studies had also revealed that, in contrast to osmium analogs, the biologically much more active ruthenium compounds are prone to reduction by ubiquitous natural reducing agents such as ascorbic acid, suggesting that they can be activated by biological nucleophiles [30].

**Table 1:** Inhibition of cancer cell growth by studied compounds in three human cancer cell lines; 50 % inhibitory concentrations (means ± standard deviations), obtained by the MTT assay and neutral red uptake assay (exposure time: 96 h)

Compounds	IC50, µM (MTT)^a^			IC50, µM (neutral red)		
	A549	CH1/PA-1	SW480	A549	CH1/PA-1	SW480
**1c**	14 ± 3	2.7 ± 0.6	2.6 ± 0.3	20 ± 8	5.7 ± 2.5	4.3 ± 1.7
**1t**	8.0 ± 1.3	1.3 ± 0.3	1.1 ± 0.3	9.3 ± 2.9	2.3 ± 0.7	1.5 ± 0.4
**2c**	128 ± 18	48 ± 13	43 ± 6	n.d.	n.d.	n.d.
**2t**	>640	145 ± 12	450 ± 35	n.d.	n.d.	n.d.

^a^Taken from Ref. [30]

The strongest difference in cytotoxicity was observed between *trans*-configured ruthenium indazole complex **1t** and its osmium analog **2t** with a maximum factor of about 400. The difference between the corresponding *cis* isomers **1c** and **2c** was also pronounced with a maximum factor of 18. In addition, the *trans*-configured ruthenium complex **1t** is up to 2.4-fold more potent than its *cis* analog **1c**, while the *cis*-configured osmium indazole complex **2c** is up to tenfold more potent than its *trans* isomer **2t**. For comparison, the antiproliferative activity of KP1019 tested previously yielded IC_50_ values of 44 ± 11 µM in CH1/PA-1 and 79 ± 5 in SW480 cells [31]. Obviously, the exchange of one indazole present in KP1019 by NO increased the cytotoxic potency of ruthenium-based nitrosyl analog **1t** about 72 times in SW480 and about 34 times in CH1/PA-1 cells.

The first cytotoxicity tests had been performed using the MTT assay, which is based on the reduction of a tetrazolium salt to an insoluble formazan, reflecting the number of viable cells present. This reduction is catalyzed by mitochondrial enzymes as well as by cytoplasmic and cell membrane components [36]. Since nitric oxide can disrupt the mitochondrial respiratory system, the NO-mediated damage of mitochondria might distort the results of MTT-based cytotoxic tests. Therefore, the activity of the most active compounds was confirmed by the neutral red uptake assay. This assay is based on the ability of viable cells to incorporate and bind the neutral red dye into lysosomes and likewise provides a quantitative estimation of the number of viable cells [37].

IC_50_ values obtained by the neutral red uptake assay are systematically slightly higher than those obtained by the MTT assay, with a maximum factor of 2.1 (for **1c** in CH1/PA-1 cells). However, these differences are too small to be taken as an indication for distortion of MTT-based cytotoxicity data by NO-mediated mitochondrial damage upon treatment with the tested substances.

**Fig. 4:**
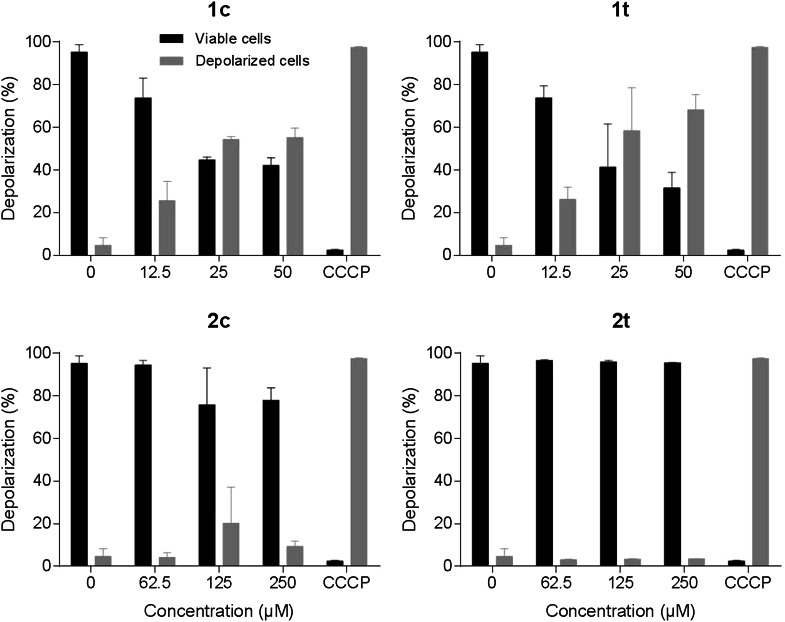
Depolarization of mitochondrial membranes in SW480 cells after 48 h exposure to the tested compounds, measured by flow cytometry using JC-1 staining. Positive control was treated with 0.9 mM CCCP

The possible influence on mitochondria was additionally investigated in SW480 cells by the lipophilic cationic dye JC-1. The JC-1 assay reveals depolarization of mitochondrial membranes in up to 55 and 68 % of cells after 48 h exposure to ruthenium-based complexes **1c** and **1t**, respectively, and up to 20 and 4 % of cells for osmium-based complexes **2c** and **2t**, respectively (Fig. 4). Thus, these experiments indicate a difference in the capacities of tested analogs to depolarize mitochondria: *trans*-configured ruthenium complex **1t** induces mitochondrial membrane depolarization to an about 17 times higher extent than its osmium analog **2t**, whereas *cis*-configured ruthenium complex **1c** does so only to an about 3 times higher extent than its osmium analog **2c**.

**Fig. 5:**
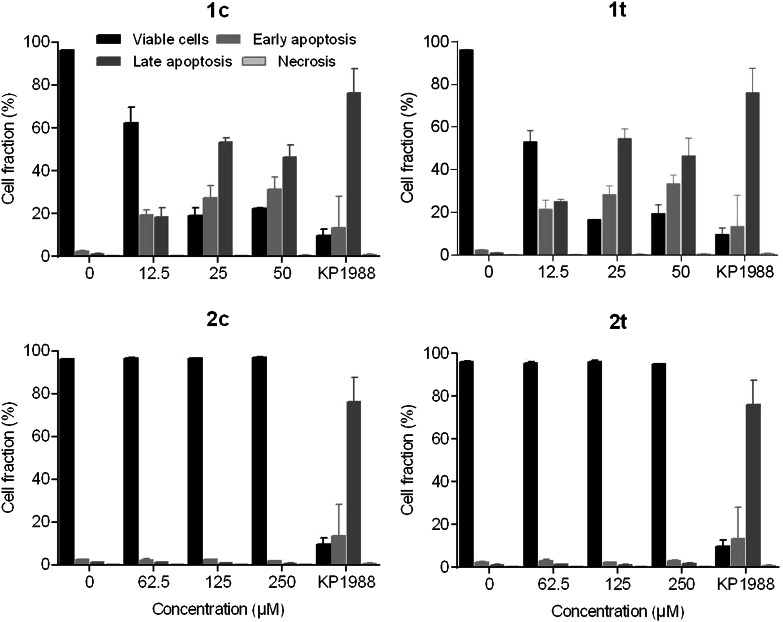
Apoptosis induction in SW480 cells after 48 h of exposure to the tested compounds, measured by flow cytometry using annexin V-FITC/propidium iodide double staining. Positive control was treated with 200 µM of platinum(II) oxime complex KP1988 [38]

Since the depolarization of mitochondrial membranes plays a role in the intrinsic apoptotic pathway, apoptosis induction was examined by annexin V-FITC/propidium iodide double staining in SW480 cells and subsequent flow cytometric analysis (Fig. 5). This assay is based on detection of phosphatidylserine externalization from the inner to the outer side of the plasma membrane upon apoptosis induction; the protein annexin V is able to bind to this externalized lipid, and the fluorescent tag FITC serves as a label for the flow cytometric detection of apoptotic cells. This is combined with propidium iodide, which indicates the loss of cell membrane integrity, differentiating necrotic and late apoptotic from early apoptotic and viable cells. These experiments indicate induction of apoptosis by ruthenium-based complexes **1c** and **1t** (50 µM) after 48 h in up to 80 and 83 % of cells, respectively, and by osmium-based complexes **2c** and **2t** (250 µM) in up to 3 and 5 % of cells. Thus, the capacity of the *cis*-configured ruthenium indazole complex **1c** to induce apoptosis is about 27 times higher than that of its osmium-based analog **2c**. The *trans*-configured ruthenium indazole complex **1t** is about 17 times more potent in this respect than its osmium analog **2t**. Together with the results of the JC-1 assay, this suggests that only ruthenium, but not osmium-based nitrosyl complexes strongly induce programmed cell death via the intrinsic pathway involving depolarization of mitochondrial membranes.

**Fig. 6:**
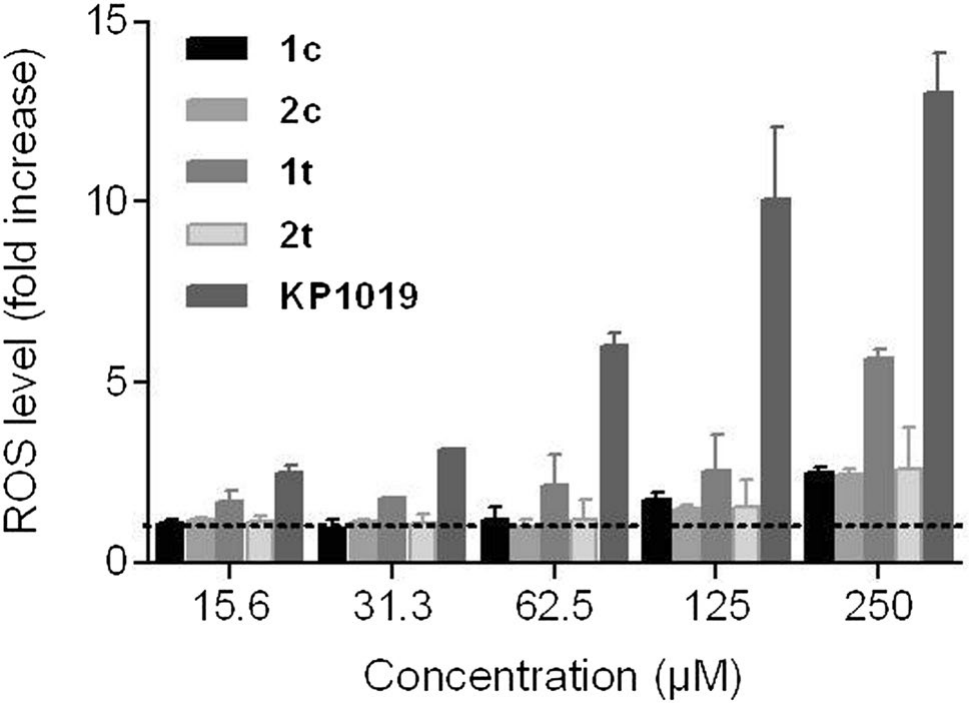
Intracellular ROS levels in HL60 cells induced by tested compounds, determined by the DCF-DA assay. RFUs of all samples were normalized to the RFU of stained untreated control (indicated by a* dashed line*)

Since the generation of oxidative stress is one of the known NO-mediated disturbances of the cell [39], we investigated by the DCF-DA assay the impact of nitrosyl complexes on the intracellular level of ROS in promyelocytic leukemia cells HL60 (Fig. 6). The assay employs the cell-permeable fluorogenic probe DCF-DA, which is rapidly oxidized to highly fluorescent DCF by ROS and its fluorescence intensity is proportional to the ROS levels within the cell. As an NO-free reference compound we used KP1019. The studies indicate a pronounced increase in ROS level by the *trans*-configured ruthenium indazole complex **1t** only at the very high concentration of 250 µM (by a factor of 5.7), whereas the reference compound KP1019 consistently induces stronger increases (by up to 13 times) and the other tested complexes only induce up to 2.5-fold increases (at 250 µM). Thus, the *trans*-configured complex KP1019 lacking an NO ligand is about 2.3 times more potently inducing ROS than the corresponding ruthenium nitrosyl complex **1t** and about 5 times more potent than the other complexes.

**Fig. 7:**
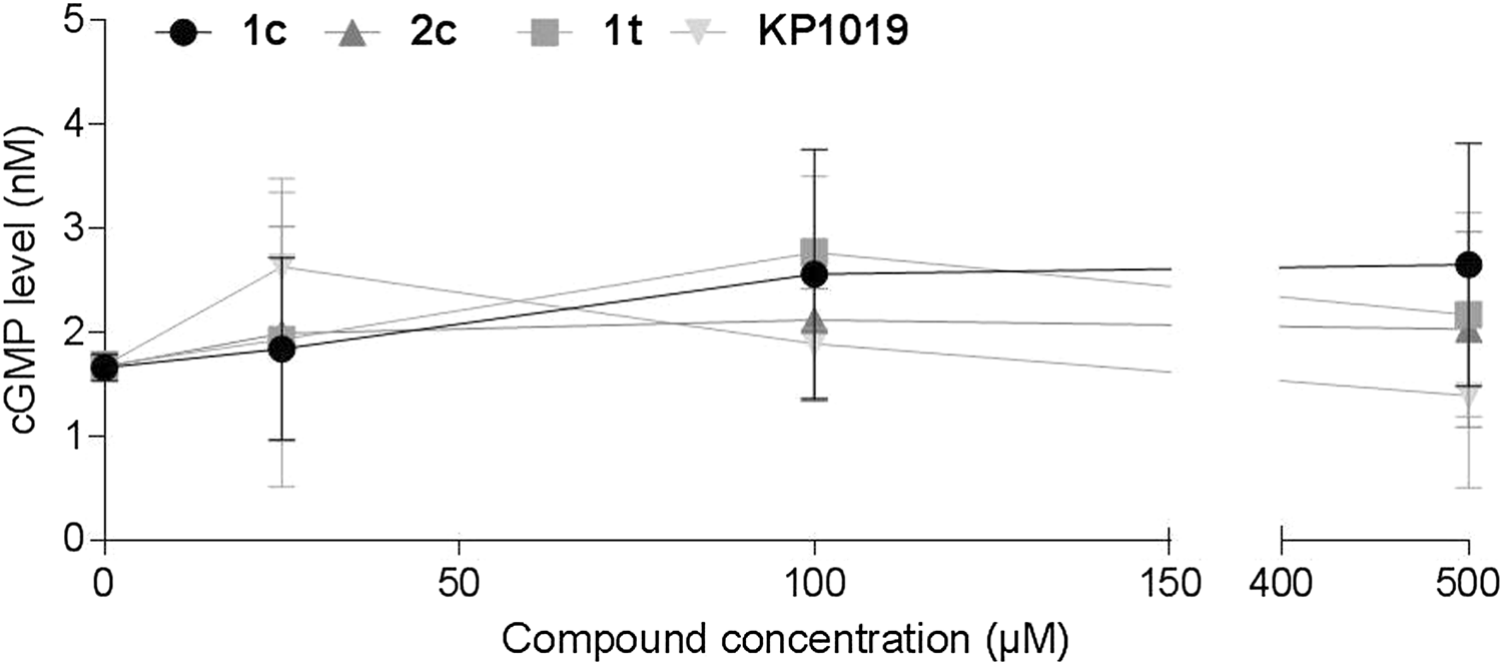
Intracellular cGMP levels in CH1/PA-1 cells upon treatment with ruthenium- and osmium-based nitrosyl complexes or KP1019, determined by the competition enzyme-linked immunoassay (cGMP assay)

The possible involvement of the cGMP pathway, as a main NO-dependent signaling pathway in the cell, was examined by the competition enzyme-linked immunoassay in CH1/PA-1 cells (Fig. 7). Because of the competitive nature of this assay, the magnitude of the absorbance is inversely proportional to the quantity of cGMP in the sample. Measurement of absorbance using a cGMP standard curve allows calculating the absolute amount of cGMP in a sample of interest. Obtained data suggest an up to 1.5-fold increase in intracellular cGMP level upon treatment with *trans*-configured ruthenium indazole complex **1t** at a concentration of 100 µM and a comparable effect of the *cis*-configured ruthenium indazole complex **1c** at the highest concentration of 500 µM, but all effects are within the ranges of standard deviations and should therefore be taken with caution.

The interactions with plasmid DNA had been studied previously for KP1019, showing its ability to untwist and to bend DNA [10]. As analogs of KP1019, the nitrosyl complexes were now studied for their ability to interact with DNA. Cell-free experiments showed no significant activity versus a dsDNA plasmid (Fig. S1). These experiments suggest that ruthenium- and osmium-based nitrosyl complexes are unable to induce DNA damage under the experimental conditions.

In summary, the metal center variation and *cis*/*trans* isomerism in nitrosyl complexes bearing azole heterocycles have an unexpectedly large impact on their potency in human cancer cell lines. Generally, ruthenium nitrosyl complexes showed a stronger capacity to inhibit cancer cell proliferation, to induce depolarization of mitochondrial membranes and apoptosis, and to increase intracellular ROS and cGMP levels than the osmium analogs. Overall, the *cis*-configured ruthenium nitrosyl complex is nearly as potent as the *trans*-configured complex except for ROS and cGMP assays.

The observed stronger activity of ruthenium complexes is remarkable and can be explained by differences in M–NO bond stabilities. There is evidence that the Os–NO bond is stronger compared to the Ru–NO analog [40]. As a consequence, the ruthenium compounds are prone to reduction in the biological environment due to the decreased stabilization of the NO ligand compared to the Os counterparts. Furthermore the ruthenium analogs can be activated by ascorbic acid, leading to hydrolysis of one chloride ligand in contrast to osmium analogs [30]. Ford has pointed out a “*trans*-effect” for ruthenium nitrosyls, which was more recently reviewed for octahedral transition metal complexes in general by Coe and Glenwright [18, 41]. Ford claims that the identity of the *trans*-ligand affects the M–NO and N–O stretching frequencies; notably, X-ray structures of ruthenium nitrosyls showed that in the *trans* position the M–ligand bond was significantly longer than in the *cis*-position. This is in line with previous research where it was shown that Ru−NO bonds in the *cis* isomer **1c** are significantly shorter than in the *trans* isomer **1t** because of the stronger *trans* effect of indazole compared to the chlorido ligand [30]. This is also confirmed by thermodynamic data showing a preference for the *cis* compound: *cis* to *trans* isomerization [−18.6 J/(mol K)] and [31.0 J/(mol K)] for the *trans* to *cis* conversion. Kinetic data reveal the same picture with rate constants of 5.51 × 10^−6^ for the *cis*→*trans* process and 12.2 × 10^−6^ for the *trans*→*cis* process at 100 °C in an aprotic solvent [34].

We come to the point that the understanding of the intracellular Ru–NO bond behavior is crucial for structure–activity relationships of this class of metallodrugs. If we take into account that the Ru–NO bond is more labile than the Os–NO bond, we may expect the release of NO under biological conditions as mentioned above. The role of NO in mitochondria-mediated cell death was reported several times before. Brown and co-workers describe a broad range of NO actions on mitochondria: it inhibits mitochondrial respiration, stimulates the production of superoxide, hydrogen peroxide and peroxynitrite, induces the transition permeability and release of cytochrome C, and NO potentially sensitizes cells to hypoxic damage [39]. Toledo et al. reported the ability of ruthenium(II) ammine nitrosyl complexes to release NO under biological conditions, where the reduction of these complexes is concomitant with mitochondrial NADH oxidation [42]. Curti and co-authors also observed NO release for this compound class which was occurring as a result of NAD(P)H oxidation and led to dissipation of mitochondrial membrane potential, ATP depletion and generation of ROS. As described in the most recent publication, oxidation of mitochondrial NADH promotes NO release from nitrosyl ruthenium complexes, which is accompanied by the release of cytochrome C [43, 44].

Thus far, our results demonstrate that the studied ruthenium nitrosyl complexes induce apoptosis by the mitochondrial pathway, at least partially associated with ROS generation, and may represent promising drug candidates for further preclinical evaluation.

## Electronic supplementary material

Below is the link to the electronic supplementary material.

Supplementary material 1 (PDF 113 kb)

## Abbreviations

CCCP: Carbonyl cyanide 3-chlorophenylhydrazone
cGMP: Cyclic guanosine monophosphate
DCFH DA: Dichloro-dihydro-fluorescein diacetate
DMSO: Dimethyl sulfoxide
FITC: Fluorescein isothiocyanate
HEPES: 4-(2-hydroxyethyl)-1-piperazineethanesulfonic acid
HRP: Horseradish peroxidase
JC-1: 5,5′,6,6′-tetrachloro-1,1′,3,3′-tetraethylbenzimidazolylcarbocyanine iodide
MTT: 3-(4,5-dimethylthiazol-2-yl)-2,5-diphenyltetrazolium bromide
NF-kB: Nuclear factor kappa-light-chain-enhancer of activated B cells
NO: Nitric oxide
PBS: Phosphate-buffered saline
PI: Propidium iodide
ROS: Reactive oxygen species
sGC: Soluble guanylate cyclase
