# Supplementary material for: Biological properties of novel ruthenium- and osmium-nitrosyl complexes with azole heterocycles
Source: J Biol Inorg Chem. 2016 Mar 9;21:347–56. doi: 10.1007/s00775-016-1345-z (PMC4850188; doi:10.1007/s00775-016-1345-z)
Supplement: Supplementary file 1 — Supplementary material 1 (PDF 113 kb) [file 775_2016_1345_MOESM1_ESM.pdf]

Supplementary information

Biological properties of novel ruthenium and osmium nitrosyl complexes with azole heterocycles

Maria S. Novak, Gabriel E. Büchel, Bernhard K. Keppler and Michael A. Jakupec

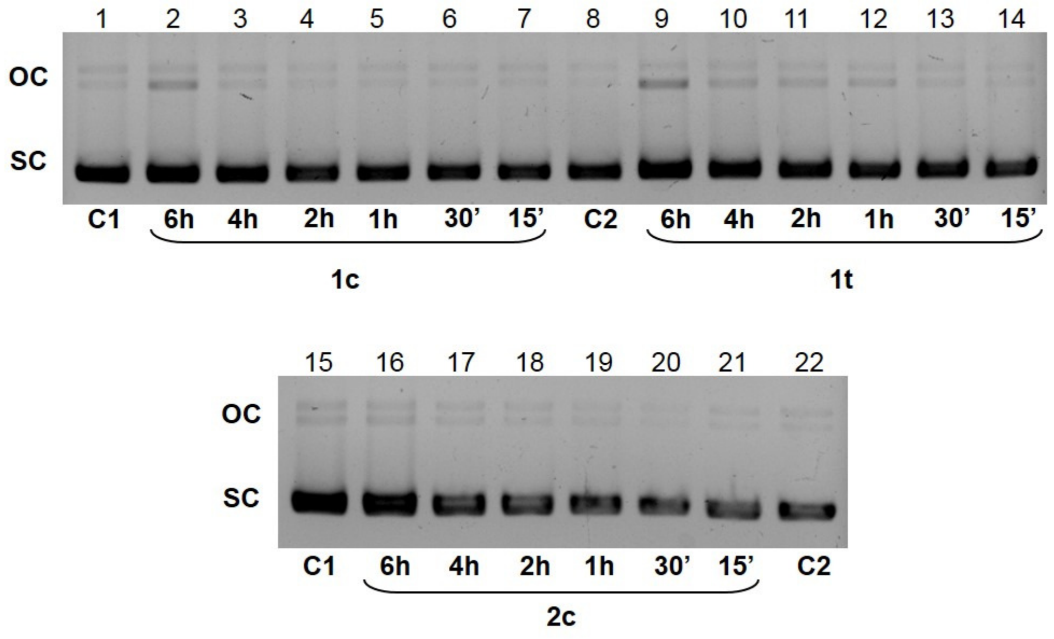

Figure S1. Electropherograms of dsDNA plasmid pUC19 after exposure to a 50 μM solution of ruthenium complexes **1c** (lanes 2–7) and **1t** (lanes 9–14), as well as osmium complex **2c** (lanes 16–21) for different exposure times (15 min to 6 h) in comparison to untreated controls C1 and C2 (lanes 1, 8, 15, 22).
